# Impact of Influenza Vaccination on Mortality in the Oldest Old: A Propensity Score-Matched Cohort Study

**DOI:** 10.3390/vaccines8030356

**Published:** 2020-07-03

**Authors:** Pauline Walzer, Clémentine Estève, Jeremy Barben, Didier Menu, Christine Cuenot, Patrick Manckoundia, Alain Putot

**Affiliations:** 1 Geriatrics Internal Medicine Department, University Hospital of Dijon Bourgogne, CEDEX, 21079 Dijon, France; pauline.walzer@gmail.com (P.W.); jeremy.barben@chu-dijon.fr (J.B.); patrick.manckoundia@chu-dijon.fr (P.M.); 2 Infectious Diseases Department, University Hospital, CEDEX, 21079 Dijon, France; clementine.esteve@chu-dijon.fr; 3 Mutualité Sociale Agricole de Bourgogne Franche Comté, 21000 Dijon, France; menu.didier@bourgogne.msa.fr (D.M.); cuenot.christine@franchecomte.msa.fr (C.C.)

**Keywords:** flu, influenza, mortality, influenza vaccination, elderly, multimorbidity, comorbidities

## Abstract

Influenza remains a major cause of illness and death in geriatric populations. While the influenza vaccine has successfully reduced morbidity and mortality, its effectiveness is suspected to decrease with age. The aim of this study was to assess the impact of influenza vaccination on all-cause mortality in very old ambulatory subjects. We conducted a prospective cohort study from 1 July 2016 to 31 June 2017 in a large unselected ambulatory population aged over 80 years. We compared all-cause mortality in vaccinated versus unvaccinated subjects after propensity-score matching, to control for age, sex and comorbidities. Among the 9149 patients included, with mean age 86 years, 4380 (47.9%) were vaccinated against influenza. In total, 5253 (57.4%) had at least one chronic disease. The most commonly vaccinated patients were those with chronic respiratory failure (76.3%) and the least commonly vaccinated were those suffering from Parkinson’s disease (28.5%). Overall, 2084 patients (22.8%) died during the study. After propensity score matching, the mortality was evaluated at 20.9% in the vaccinated group and 23.9% in the unvaccinated group (OR = 0.84 [0.75–0.93], *p* = 0.001). This decrease in mortality in the vaccinated group persisted whatever the age and Charlson Comorbidity index. In conclusion, nearly a half of this ambulatory elderly population received Influenza vaccine. After adjustment on comorbidities, influenza vaccination was associated with a significant decrease in all-cause mortality, even in the eldest multimorbid population. Improving immunization coverage in this frail older population is urgently needed.

## 1. Introduction

Influenza is a leading cause of hospitalization and death in older patients [1,2]. Every year, 290,000–650,000 people die from influenza worldwide [3,4] and 90% of these deaths occur among older adults [1,5]. In addition to the prevention of infection-related morbidity and mortality, it is now well established that influenza vaccine has a protective effect on decompensation due to underlying diseases, especially cardiovascular diseases [6,7]. Several studies have assessed the effectiveness of the influenza vaccine in preventing influenza illness and hospital admissions [8,9,10,11]. Additional recent studies have confirmed a reduction in all-cause mortality as well as influenza-related hospitalizations in elderly vaccinated individuals [12,13].

However, influenza vaccine effectiveness may decrease with advanced age [8] and the effect of vaccination on deaths from any cause is not confirmed [14]. Despite an increase in immunization coverage over time, the percentage of influenza-related deaths in winter is stable at around 5–10% [5,15]. Furthermore, vaccine efficacy is thought to be reduced in older age due to immunosenescence [16], inadequate immunization coverage and inappropriate vaccine formulation for the specific immune profile of older subjects [17]. In observational studies, comorbidities appear as confounding factors between influenza vaccination and mortality and should be more investigated to reduce biases and identify the most at-risk populations [18,19,20]. To assess the impact of vaccination on all-cause mortality, we conducted a prospective cohort study in a community-living population aged over 80 years.

## 2. Materials and Methods

### 2.1. Study Design

This prospective cohort study was conducted from 1 July 2016 to 31 June 2017, from the *Mutualité Sociale Agricole de Franche-Comté* (MSA) database. MSA is a French regional health insurance plan for active or retired agricultural workers that covers health costs, daily allowances and carries out preventive actions. This database prospectively records all prescriptions and chronic diseases reported by the referring physician, as these conditions (*Affection Longue Durée*) are fully covered by the French health insurance, leading to a systematic reimbursement of related health expenses.

The present study complied with the Declaration of Helsinki. Data were anonymously managed. No consent was required.

### 2.2. Patients

All patients registered in MSA and aged over 80 years were included and then divided into two groups: a group of patients for whom a prescription of influenza vaccine was delivered during the period of the study (vaccinated group) and a group with no influenza vaccine prescription (unvaccinated group).

### 2.3. Data Collection

Age, gender and chronic diseases (according to the International Statistical Classification of Diseases and Related Health Problems—10th edition (ICD-10)), Charlson Comorbidity Index (CCI) [21] and vital status at the end of the study period were recorded for all patients. Multimorbidity was defined as a CCI ≥ 5.

Pneumococcal vaccination and antibiotic prescription during the period of study were also collected.

### 2.4. Outcome

The primary outcome was adjusted all-cause mortality during the study period.

### 2.5. Statistical Analyses

Vaccinated and unvaccinated groups were compared using univariate and multivariable analysis. Continuous variables were expressed as medians and interquartile ranges. A Kolmogorov–Smirnov test was performed to analyze the normality of continuous variables. The Student *t*-test or the Mann–Whitney test was used to compare continuous variables, and the Chi2 or Fisher test was used to compare dichotomous data, as appropriate.

Given the non-randomized design of the study, we used a propensity score (PS) to identify and control for confounding factors that could influence the likelihood of vaccination. A multivariable logistic regression model was built to estimate vaccination risk and calculate the PS for vaccination. The variables included in the multivariable model with a threshold at 5% were: age, gender, stroke, peripheral arterial disease, heart failure, diabetes, hypertension, coronary artery disease, respiratory failure, Parkinson’s disease, chronic kidney disease, neoplasia, other chronic diseases, number of chronic diseases and CCI. 

The patients with and without vaccination were then paired 1:1 on this PS using a caliper width of 5% of the standard deviation of the PS logit. 

We used the SPSS 13.0 software package (IBM Corp., Armonk, N.Y., USA) for all analyses.

## 3. Results

### 3.1. Patients Characteristics

Among the 9149 patients included, the median age was 86.5 years [83.5–90.2]; women represented 60.9% of the population.

For vaccination status, 47.9% were vaccinated against influenza and 2.5% against pneumococcal infections. The characteristics of the subjects vaccinated against influenza and unvaccinated, before and after matching, are presented in Table 1. Concerning comorbidities, 57.4% patients had at least one or more chronic diseases. The most common chronic diseases were: chronic heart failure (19.8%), diabetes (11.0%), cancer (10.7%), coronary artery disease (8.1%), cognitive disorders (6.5%), peripheral arterial disease (5.7%) and stroke (4.3%). Multimorbidity (CCI ≥ 5) was identified in 58.7% of patients.

### 3.2. Vaccination Coverage

**Figure 1:**
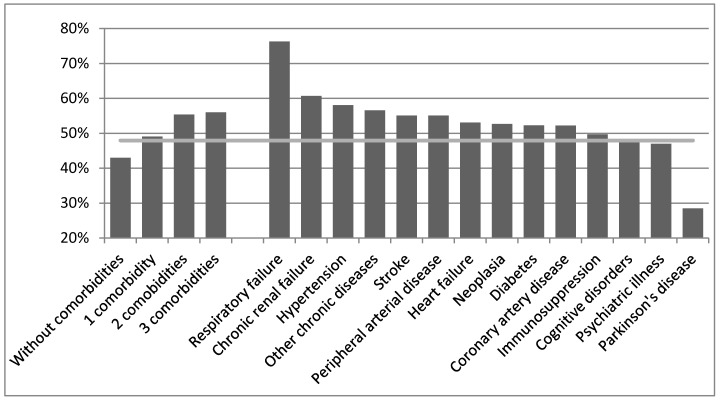
Influenza vaccine coverage according to comorbidities during the 2016–2017 season. The grey line indicates the mean rate of vaccination in the whole population.

The total influenza vaccination coverage of the study population was 47.9%. The most commonly vaccinated subjects were those with chronic respiratory failure (76.3%) and those with chronic renal failure (60.7%). The least commonly vaccinated were those with Parkinson’s disease (28.5%). The results of influenza vaccine coverage by group of chronic conditions and by number of comorbidities are presented in Figure 1.

### 3.3. Mortality

**Figure 2:**
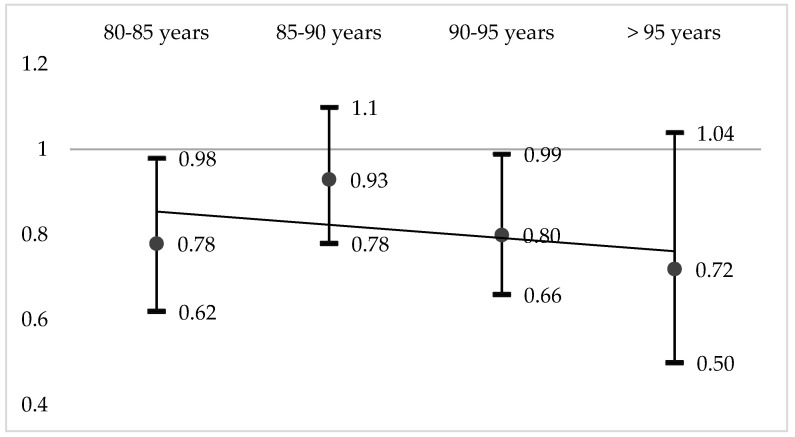
Adjusted odds ratio (95% Confidence interval) of risk of mortality in vaccinated versus non-vaccinated groups according to age (trend line in grey).

**Figure 3:**
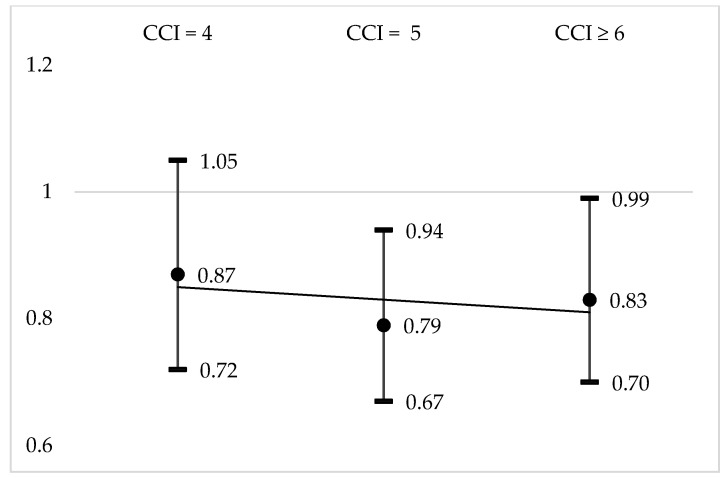
Adjusted odds ratio (95% Confidence interval) of risk of mortality in vaccinated versus non-vaccinated groups according to Charlson Comorbidity Index (CCI) (trend line in grey).

In total, 2084 subjects (22.8%), mean age 89.6 years, died during the study period. After matching on the propensity score, mortality was 20.9% in the vaccinated group and 23.9% in the unvaccinated group (OR = 0.84 [0.75–0.93], *p* = 0.001). Adjusted odds ratios of risk of mortality in vaccinated versus non-vaccinated groups according to age and CCI classes are presented in Figure 2 and Figure 3, respectively. As highlighted, mortality remained lower whatever the age and comorbidities in the vaccinated group compared to the non-vaccinated group.

## 4. Discussion

Older adults and those with underlying comorbidities, including chronic cardiovascular and lung diseases, immunosuppression and diabetes, have a higher risk of hospitalization and severe disease, and are consequently priority groups for influenza vaccination [22]. However, in our oldest multimorbid population at the highest risk of influenza complications, less than a half was vaccinated. The WHO goal of vaccinating 75% of high-risk patients was achieved only for the subgroup of patients with chronic respiratory failure.

However, our study suggests that influenza vaccination is associated with lower all-cause mortality in very elderly and comorbid subjects. Our results are consistent with those of other studies. In particular, Voordouw et al. showed that annual influenza vaccination and revaccination were associated with a reduction in all-cause mortality risk, similarly in healthy and comorbid subjects of 80 years and older (hazard ratio 0.81, 95% interval confidence: 0.66–1.00; and 0.69, 0.61–0.78, respectively) [23]. In accordance with our results, a recent study in an elderly Italian population highlighted that influenza vaccine was equally effective for death prevention in all age groups (≤74, 75–84 and ≥85 years old) [24]. Other authors, in a large cohort study of subjects over 65 years of age, noted a decrease in mortality of 14%, 19% and 1% associated with vaccination during three consecutive winter seasons between 1998 and 2001 [25]. Nevertheless, some other studies, after adjustment by age and type of circulating virus, failed to highlight any decrease in influenza-related excess mortality per year, despite an increase in vaccination coverage over time [5,15]. This implies an overestimation of vaccination benefit in the very elderly and the existence of bias in observational studies evaluating vaccine efficacy. 

Currently, for ethical reasons, only observational studies can assess the effectiveness of influenza vaccine within a population. Nevertheless, one randomized placebo-controlled trial was carried out during the 1991–1992 season in patients over the age of 60 in Holland [8]. In this study, vaccine efficacy was 50% (95% CI 39–65%) against influenza confirmed by serology, but only 23% (95% CI 51–61%) in subjects 70 years and over, suggesting a decrease in efficiency with increasing age. More recently, meta-analyses have evaluated an influenza vaccine efficacy of 50% (95% CI 45–56%) and 47% (95% CI 39–54%) to prevent all-cause deaths in subjects 65 and over [10,11]. By contrast, the Cochrane Database of Systematic Reviews published a meta-analysis in 2018 showing no reduction in mortality from influenza or all causes in elderly subjects vaccinated against influenza (RR 1.02, 95% CI 0.11–9.72) [14]. Additionally, authors have highlighted the existence of biases since they observed a greater reduction in the risk of death from all causes before the influenza season when there should be no vaccine effect [26,27], assuming preferential reception of the vaccine by healthy subjects.

The influenza vaccination coverage of our population was comparable to those observed in the elderly in France (50%) [28,29] and Europe (47.1%) [30] in the same year. This rate remains far below the target of 75% vaccination coverage recommended by the World Health Organization [22]. The flu epidemic strain in France during 2016–2017 was A(H3N2) at 98% and was covered by all the influenza vaccines prescribed.

Our results indicate that certain comorbidities associated with higher or lower rates of vaccine coverage. Subjects with respiratory failure were more vaccinated against influenza than the rest of the study population. Other reports also highlight higher vaccination coverage in pulmonary diseases compared with other groups at risk [28]. Patients with respiratory failure or heart disease appear to have the highest risk of mortality attributable to influenza [20]. Conversely, subjects with Parkinson’s disease were significantly less vaccinated than the rest of the study population. Interestingly, lower vaccination rates have already been reported in patients with neurodegenerative disorders compared with other patients [31,32]. We can assume that practitioners are more likely to abandon preventive for palliative care, limited to symptom reduction, in patients with neurocognitive disorders, as observed in cardiovascular care [33].

Even though vaccine effectiveness seems to decrease in frail subjects [34,35], similar to other authors [23], we observed a persistent lower risk of mortality in vaccinated patients with multimorbidity (CCI > 5) compared with non-vaccinated patients.

Some limitations should be mentioned. First, as for all observational studies, it cannot be excluded that the decrease in mortality observed in the vaccinated group was linked to confounding factors. To minimize these biases, we performed a propensity score analysis. Nevertheless, certain characteristics such as performance status, socioeconomic status, body mass index, smoking and alcohol consumption were not collected, even though they are known to be factors associated with higher mortality. In particular, material deprivation and low access to health care services are associated with a lower frequency of influenza vaccination [36]. Unfortunately, as the date of death was not recorded in this series, we were unable to compare mortality in vaccinated and non-vaccinated groups before the flu season, as suggested by others to assess the existence of biases [27]. Moreover, it remains undetermined for which causes of death influenza vaccination could be associated with a protective effect in this study. Second, only chronic diseases were recorded in the present study, although acute diseases also have a major impact on mortality. Third, only vaccine prescriptions and not effective administrations were recorded. Finally, our population is mostly composed of retired French farmers and it remains uncertain that these results can be extrapolated to the general population.

## 5. Conclusions

Notwithstanding its observational design, this study suggests that influenza vaccination does have a benefit for very elderly people. More specifically, it appears to reduce all-cause mortality, whatever the age and comorbidities. Patient comorbidities were associated with highly variable vaccination rates: subjects with chronic respiratory disease were the most vaccinated and those suffering from neurodegenerative disorders were the least vaccinated from this cohort of older patients. Vaccination coverage needs to be urgently improved in this very-high-risk population.

## Figures and Tables

**Table 1:** Comparison of vaccinated and unvaccinated subjects against influenza before and after matching.

	Before Matching			After Matching		
Characteristics	Vaccinated	Unvaccinated	*p*	Vaccinated	Unvaccinated	*p*
	*n* = 4380	*n* = 4769		*n* = 3935	*n* = 3935	
**Age**	86.5 [83.5–90.3]	86.5 [83.5–90.1]	0.8	86.6 [83.5–90.1]	86.5 [83.5–90.2]	0.7
80–90 years	3248 (74.2)	3488 (73.1)	0.3	2919 (74.2)	2891 (73.5)	0.5
>90 years	1132 (25.8)	1281 (26.9)	0.3	1016 (25.8)	1044 (26.5)	0.5
**Sex**						
Female	2538 (57.9)	3038 (63.7)	<0.001	2356 (59.9)	2348 (59.7)	0.9
Male	1842 (42.1)	1731 (36.3)	<0.001	1579 (40.1)	1587 (40.3)	0.9
**Pneumococcal vaccination**	154 (3.5)	74 (1.6)	<0.001	83 (2.1)	73 (1.9)	0.4
**Chronic disease**						
Stroke	218 (5.0)	178 (3.7)	0.003	165 (4.2)	159 (4.0)	0.7
Peripheral arterial disease	287 (6.6)	234 (4.9)	0.001	227 (5.8)	227 (5.8)	1.0
Heart failure	962 (22.0)	849 (17.8)	<0.001	817 (20.8)	769 (19.5)	0.2
Chronic liver disease	4 (0.1)	6 (0.1)	0.6	4 (0.1)	5 (0.1)	0.7
Diabetes	528 (12.1)	481 (10.1)	0.003	453 (11.5)	437 (11.1)	0.6
Hypertension	223 (5.1)	161 (3.4)	<0.001	171 (4.3)	161 (4.1)	0.6
Coronary artery disease	387 (8.8)	354 (7.4)	0.01	343 (8.7)	337 (8.6)	0.8
Respiratory failure	151 (3.4)	47 (1.0)	<0.001	57 (1.4)	46 (1.2)	0.3
Cognitive disorders	282 (6.4)	309 (6.5)	0.9	261 (6.6)	280 (7.1)	0.4
Parkinson’s disease	45 (1.0)	113 (2.4)	<0.001	44 (1.1)	28 (0.7)	0.06
Chronic kidney disease	71 (1.6)	46 (1.0)	0.005	52 (1.3)	46 (1.2)	0.5
Psychiatric disorders	62 (1.4)	70 (1.5)	0.8	56 (1.4)	56 (1.4)	1.0
Neoplasia	516 (11.8)	464 (9.7)	0.002	443 (11.3)	431 (11.0)	0.7
Other chronic diseases	197 (4.5)	151 (3.2)	0.001	150 (3.8)	149 (3.8)	1.0
Other neurological diseases	31 (0.7)	32 (0.7)	0.8	28 (0.7)	29 (0.7)	0.9
Immunosuppression	87 (2.0)	88 (1.8)	0.6	74 (1.9)	76 (1.9)	0.9
**Number of chronic diseases**						
0	1677 (38.3)	2219 (46.5)	<0.001	1633 (41.5)	1690 (42.9)	0.5
1	1651 (37.7)	1709 (35.8)	<0.001	1459 (37.1)	1451 (36.9)	0.5
2	782 (17.9)	629 (13.2)	<0.001	643 (16.3)	602 (15.3)	0.5
3	290 (6.2)	212 (4.4)	<0.001	200 (5.1)	192 (4.9)	0.5
**Charlson Comorbidity Index**	5 [4,5]	5 [4,6]	<0.001	5 [4,5]	5 [4,6]	0.8
<5	1696 (38.7)	2087 (43.8)	<0.001	1625 (41.3)	1612 (41.0)	0.8
≥5	2684 (61.3)	2682 (56.2)	<0.001	2310 (58.7)	2323 (59.0)	0.8
**Deaths**	935 (21.3)	1149 (24.1)	0.002	827 (21.0)	943 (24.0)	0.002
